# Uncovering interactions between mycobacterial respiratory complexes to target drug-resistant *Mycobacterium tuberculosis*

**DOI:** 10.3389/fcimb.2022.980844

**Published:** 2022-08-24

**Authors:** Matthew B. McNeil, Chen-Yi Cheung, Natalie J. E. Waller, Cara Adolph, Cassandra L. Chapman, Noon E. J. Seeto, William Jowsey, Zhengqiu Li, H. M. Adnan Hameed, Tianyu Zhang, Gregory M. Cook

**Affiliations:** ^1^ Department of Microbiology and Immunology, University of Otago, Dunedin, New Zealand; ^2^ Maurice Wilkins, Centre for Molecular Biodiscovery, The University of Auckland, Auckland, New Zealand; ^3^ School of Pharmacy, Jinan University, Guangzhou, China; ^4^ State Key Laboratory of Respiratory Disease, Guangzhou Institutes of Biomedicine and Health (GIBH), Chinese Academy of Sciences (CAS), Guangzhou, China; ^5^ China-New Zealand Joint Laboratory of Biomedicine and Health, Guangzhou Institutes of Biomedicine and Health (GIBH), Chinese Academy of Sciences (CAS), Guangzhou, China; ^6^ Guangdong-Hong Kong-Macau Joint Laboratory of Respiratory Infectious Diseases, Guangzhou, China; ^7^ University of Chinese Academy of Sciences (UCAS), Beijing, China

**Keywords:** respiration, synthetic lethality, drug combinations, antibiotics, synergy

## Abstract

*Mycobacterium tuberculosis* remains a leading cause of infectious disease morbidity and mortality for which new drug combination therapies are needed. Mycobacterial bioenergetics has emerged as a promising space for the development of novel therapeutics. Further to this, unique combinations of respiratory inhibitors have been shown to have synergistic or synthetic lethal interactions, suggesting that combinations of bioenergetic inhibitors could drastically shorten treatment times. Realizing the full potential of this unique target space requires an understanding of which combinations of respiratory complexes, when inhibited, have the strongest interactions and potential in a clinical setting. In this review, we discuss (i) chemical-interaction, (ii) genetic-interaction and (iii) chemical-genetic interaction studies to explore the consequences of inhibiting multiple mycobacterial respiratory components. We provide potential mechanisms to describe the basis for the strongest interactions. Finally, whilst we place an emphasis on interactions that occur with existing bioenergetic inhibitors, by highlighting interactions that occur with alternative respiratory components we envision that this information will provide a rational to further explore alternative proteins as potential drug targets and as part of unique drug combinations.

## Introduction

*M. tuberculosis*, the causative agent of tuberculosis (TB), is an obligate human pathogen and significant cause of infectious disease morbidity and mortality, being responsible for an estimated 5.8 million new infections and 1.3 million deaths among HIV-negative people and an additional 214 000 among HIV-positive people (WHO, 2021). Combination drug regimens are favored for the treatment of TB due to their ability to increase efficacy, delay the development of resistance and reduce toxic side effects. Favorable drug interactions are typically the result of either (I) therapeutic synergy, when the effect (i.e. growth inhibition or bacterial killing) of drug combinations is greater than the sum of the individual drugs, or (II) synthetic lethal interactions, when the individual drugs alone are non-lethal, whilst the combination results in killing. Alternatively, some drug combinations serve to prevent the emergence of drug resistance, with additive drugs preventing the isolation of resistant mutants despite not increasing the killing of the partner compound. The need for combination therapies for the treatment of *M. tuberculosis* is also necessitated by additional factors including (I) the unique cellular structure of *M. tuberculosis* that makes it inherently tolerant of many antibiotics, (II) the ability of *M. tuberculosis* to switch into a metabolically inactive state that is phenotypically tolerant to many antibiotics and host-immune responses, and (III) unequal drug penetration at the site of infection (Kerantzas and Jacobs, 2017). As a result, drug-susceptible strains of *M. tuberculosis* are treated with a frontline combination regimen of four antibiotics for two months followed by four months of isoniazid and rifampicin. Typically the frontline regimen achieves cure rates of 85%. Unfortunately, the prolonged treatment time of available regimens can lead to patient non-compliance, which ultimately drives the acquisition of drug resistance. Consequently, despite the extensive use of combination therapies, there is clinical resistance to all available antibiotics. Treatment options for drug resistant strains are limited, often highly toxic and require even longer treatment times, typically >9 months (Lange et al., 2018), with much lower cure rates. Whilst new regimens, including the BPaL regimen, can reduce treatment length of drug-resistant strains to 6-months, there remains an urgent need for new drug classes and new approaches to the design of combination therapies to prevent drug resistance and further reduce treatment times.

The discovery and subsequent FDA approval of the F_1_F_o_-ATP synthase inhibitor bedaquiline (BDQ) has shifted the focus of many drug-discovery programs towards the development of bioenergetic inhibitors i.e., compounds that target the mycobacterial electron transport chain (ETC) (Andries et al., 2005; Diacon et al., 2014; Cook et al., 2017). The mycobacterial ETC is a series of membrane-bound or membrane-associated enzymes that are responsible for coupling the oxidation of electron donors that are generated from central carbon metabolism to the reduction of oxygen as a terminal electron acceptor. Several of these enzymes are proton pumping, allowing for the establishment of a proton motive force (PMF) that is used to generate ATP *via* oxidative phosphorylation (Cook et al., 2017; Hards et al., 2020). Several recent studies have highlighted the benefit of combining bioenergetic inhibitors to accelerate the rate of bacterial clearance from various infection models. More importantly, the clinical efficacy of multiple bioenergetic inhibitors is highlighted by the FDA approval of the BPaL combination therapy that consists of two bioenergetic inhibitors and one translational inhibitor (Conradie et al., 2020).

The focus of this review is to highlight recent advances in our understanding of interactions between bioenergetic components and their associated inhibitors. We discuss interactions identified (either synergistic or antagonistic) from chemical, genetic and chemical-genetic and interaction studies. Whilst each strategy has drawbacks, each approach has contributed novel insights.

## Respiratory components as targets for bioenergetic inhibitors in *M. tuberculosis*

Here for context, we provide a brief description of each component that forms the mycobacterial ETC. Whilst the majority of this context is based on results from *M. tuberculosis*, we do highlight results obtained from the fast-growing model organism *Mycobacterium smegmatis*. We acknowledge that there may be differences between these species and have highlighted this when it may be a confounding factor. Prior reviews should be consulted for a more detailed discussion of each component and their functions (Cook et al., 2017; Hards and Cook, 2018; Hards et al., 2020; Hasenoehrl et al., 2020).

## Mycobacterial electron donating dehydrogenases

The mycobacterial ETC has several primary dehydrogenases that couple the oxidation of respiratory substrates or recycling of redox cofactors from the citric acid cycle to the reduction of the electron carrier menaquinone. Dehydrogenases that donate electrons to the ETC, including glycerol-3-phosphate dehydrogenase, carbon-monoxide dehydrogenase, proline dehydrogenase or L-lactate dehydrogenase are not directly linked to the citric acid cycle and are not discussed in this review, although they are expertly reviewed elsewhere (Rhee et al., 2011; Cook et al., 2014; Cook et al., 2017; Hasenoehrl et al., 2020).

**Figure 1:**
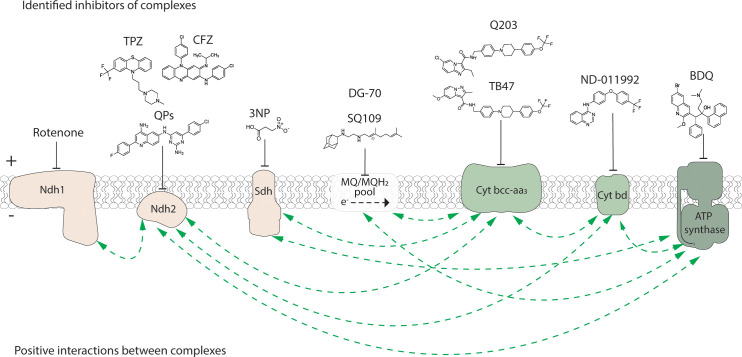
Inhibitors of the electron transport chain and ATP synthase of M. tuberculosis. Known inhibitors of respiratory components, example structures and their targets are illustrated with flathead arrows. Flathead arrow placement does not reflect the specific site of inhibition. The dashed arrow in the MQ/MQH2 pool represents the direction of electron flow. Electron transport chain complexes and the ATP synthase are coloured as: election donors, beige; electron acceptors, green. Plus (+) and minus (-) symbols represent high and low proton concentration gradient across the cytoplasmic membrane, respectively. QPs, quinolinyl pyrimidines; TPZ, trifluoperazine; CFZ, clofazimine; 3NP, 3-nitropropionate; BDQ, bedaquiline. Positive interactions (i.e. synergy or synthetic lethality) between bioenergetic components as reported from either a chemical, genetic or chemical-genetic interaction study and as described in the body of the manuscript are shown with dashed green arrows.

*M. tuberculosis* has two classes of NADH: menaquinone oxidoreductases that couple the oxidation of NADH to the reduction of menaquinone (Weinstein et al., 2005; Yano et al., 2006). This includes a proton-pumping type I NADH dehydrogenase (NDH-1) and a non-proton pumping type II NADH dehydrogenase (NDH-2) (**Figure 1**). Prior work has demonstrated that NDH-1, is analogous to mitochondrial Complex-1 and is not required for the growth of *M. tuberculosis* (Rao et al., 2008; Heikal et al., 2014; Heikal et al., 2018; Petri et al., 2018; Vilcheze et al., 2018; Beites et al., 2019). *M. tuberculosis* has two copies of NDH-2, encoded by *ndh* and *ndhA* (Vilcheze et al., 2018). Genetic studies have demonstrated that *ndh* and *ndhA* can be individually deleted, but cannot be simultaneously deleted when grown in the presence of fatty acids (Vilcheze et al., 2018; Beites et al., 2019). Growth in the absence of fatty acids, restores this conditional essentiality (Beites et al., 2019). Somewhat unexpectedly, and in contrast to prior chromosomal deletion mutants, the use of CRISPR interference demonstrated that the transcriptional repression of *ndh*, but not *ndhA*, is sufficient to impair the growth of *M. tuberculosis* (Bosch et al., 2021; McNeil et al., 2021). Furthermore, the transcriptional repression of *ndh* resulted in bacterial killing in both *M. tuberculosis* and *Mycobacterium smegmatis*, a fast-growing non-pathogenic mycobacterial species (McNeil et al., 2022). The discrepancies in phenotypes associated with the genetic inhibition of *ndh* are likely to be a result of differences in experimental approaches with chromosomal deletion mutants selecting for metabolically adapted strains that are able to grow in the absence of *ndh*, whilst transcriptional inhibition may observe the immediate effects of inhibiting *ndh* prior to metabolic adaptation (Weinstein et al., 2005; Yano et al., 2006; Heikal et al., 2016).

Two mycobacterial succinate dehydrogenase enzymes (i.e., SDH-1 and SDH-2) directly link the citric acid cycle to the ETC, by coupling the oxidation of succinate to the reduction of menaquinone in mycobacterial species (Berney and Cook, 2010; Pecsi et al., 2014; Heikal et al., 2016; Hards et al., 2019; Hards et al., 2020). Prior genetic strategies in *M. tuberculosis* have demonstrated that each complex can be individually deleted and are functionally redundant as a SDH-1 and SDH-2 double mutant was non-viable (Hartman et al., 2014). Interestingly, deletion of SDH-1 in *M. tuberculosis* disrupted cellular respiration, accelerated cell death during stationary phase and had a survival defect during chronic infection (Hartman et al., 2014). SDH-2 was essential in *M. smegmatis* (Pecsi et al., 2014), further supporting the hypothesis that these enzymes perform unique, yet overlapping functions. Definitive evidence that SDH enzymes are indeed important for survival and persistence come from the study of the succinate dehydrogenase suicide inhibitor 3-nitropropionate (3NP) (Eoh and Rhee, 2013). 3NP is a valuable tool for *in vitro* studies of SDH, but cannot be used to assess the chemical inhibition of SDH in an infection model because of its associated toxicities. Inhibiting *M. tuberculosis* SDH (SDH-1 or 2 or both) using 3NP resulted in a time-dependent killing of *M. tuberculosis* during adaptation to hypoxia/persistence (Eoh and Rhee, 2013). Re-aeration of these cultures in the presence of 3NP also inhibited growth. Treatment of non-replicating/hypoxia cultures of *M. smegmatis* with 3NP leads to cell death *via* a mechanism that dissipates the membrane potential (Pecsi et al., 2014). Taken together these studies establish that sustained metabolism of succinate through SDH is an essential component of *M. tuberculosis* metabolic adaptation to hypoxia/persistence and that SDH inhibitors will target these cells. Resolving the mechanisms of functional redundancy and specific functions of each SDH enzyme in combination with recently published structures will assist in the design of chemical inhibitors (Gong et al., 2020; Zhou X. et al., 2021).

Like SDH enzymes, mycobacterial malate:quinone oxidoreductase (MQO) couples the oxidation of malate as part of the citric acid cycle to the reduction of menaquinone (Harold et al., 2022). *M. tuberculosis* also encodes a malate-dehydrogenases (MDH) that couples the oxidation of malate to the recycling of NAD^+^ to NADH. CRISPR interference of both *mdh* and *mqo* expression in *M. tuberculosis* impairs growth more severely than either gene knockdown alone (Harold et al., 2022). It has been proposed that exergonic MQO activity powers mycobacterial growth under non-energy limiting conditions, and that endergonic MDH activity complements MQO activity, but at an energetic cost for mycobacterial growth (Harold et al., 2022). *M. tuberculosis* utilises MDH to facilitate a reductive TCA cycle to produce fumarate (oxaloacetate reduction) for use as a terminal electron acceptor (Watanabe et al., 2011). This provides an alternative electron acceptor for *M. tuberculosis* in conditions of hypoxia thereby providing a mechanism to maintain the membrane potential and recycle reducing agents. *M. tuberculosis* can also use Mqo and Mdh in combination to form a futile cycle to provide a pseudo NDH-2-type enzyme that is able to complement the growth of *ndh2* mutants (Miesel et al., 1998; Vilcheze et al., 2005). Rittershaus et al. (2018) demonstrated that MDH is required for the metabolism and survival of *M. tuberculosis in vitro* and *in vivo*. Moreover, chemical inhibitors of *M. tuberculosis* MDH had the ability to inhibit growth and rapidly kill hypoxic quiescent cells (non-replicating) *in vitro* and during infection of the murine lung (Rittershaus et al., 2018). We propose that the addition of an inhibitor of MQO, in combination with MDH inhibitors, may enhance the growth inhibition of *M. tuberculosis* further while still retaining potent activity against non-replicating populations. In this regard, MQO is not found in mammalian cells and potent inhibitors have been reported that target the mitochondrial malate:quinone oxidoreductase of the malarial parasite *Plasmodium falciparum* (Hartuti et al., 2018). The significant structural differences reported between the human and mycobacterial MDH enzymes (Rittershaus et al., 2018) suggest a combination therapy of MQO and MDH inhibitors may be attractive for TB drug development.

## Mycobacterial electron carriers

Menaquinone is the primary electron carrying lipoquinone in mycobacteria that is responsible for shuttling electrons between respiratory dehydrogenases and terminal oxidases (Cook et al., 2017). Menaquione biosynthesis occurs *via* a series of enzymes that are similar to the *E. coli* synthesizing genes *menA-J* (Cook et al., 2017). Despite the importance of menaquinone biosynthesis in ETC function, not all menaquinone synthesizing genes are designated as essential genes (Dhiman et al., 2009; Griffin et al., 2011; Cook et al., 2017; Bosch et al., 2021; McNeil et al., 2021). Interestingly, targeted transcriptional repression of *menA* and *menD* with CRISPR interference had no effect on mycobacterial growth (McNeil et al., 2021). This is in contrast to transposon mutagenesis and whole genome CRISPR-interference studies, in which both genes were designated as being necessary for mycobacterial growth (Griffin et al., 2011; Bosch et al., 2021). Prior studies have demonstrated that the consequences of CRISPRi mediated inhibition of individual metabolic genes can be mitigated by metabolic buffering, in which multiple cell divisions are needed to deplete enzymes or enzyme products below levels needed to produce a bacterial phenotype (Donati et al., 2021). Whether this is the case for menaquione biosynthesis genes in *M. tuberculosis* requires further investigation. Nevertheless, the absence of menaquinone from humans, makes it an attractive drug target. Multiple small molecules have been identified as inhibitors of various menaqinone biosynthetic enzymes including MenA and MenD (Dhiman et al., 2009; Fang et al., 2010; Debnath et al., 2012; Berube et al., 2019; Bashiri et al., 2020). Further to this, recent metagenomic guided drug discovery efforts recently identified novel antibiotic classes that directly bind menaquinone, several of which had *in vitro* and *ex vivo* activity against *M. tuberculosis* (Li et al., 2022).

## Electron accepting terminal oxidases

Under aerobic conditions, a supercomplex of cytochrome *bc*_1_ (Complex III; *qcrBCD*) and an *aa*_3_-type cytochrome *c* oxidase (Complex IV; *ctaBCDE*), herein referred to as Cyt-*bc*_1_ *aa*_3_, couples the transfer of (i) electrons from reduced menaquinol to cytochrome *c* to (ii) proton translocation and the generation of a PMF (Gong et al., 2018; Wiseman et al., 2018). Both complexes are essential for mycobacterial growth, yet the deletion or transcriptional inhibition of either complex in *M. tuberculosis* results in a bacteriostatic phenotype (Beites et al., 2019; Bosch et al., 2021; McNeil et al., 2021; McNeil et al., 2022). Numerous small molecule inhibitors of cytochrome *bc1* have been identified (Kang et al., 2014; Lu et al., 2019; Yu et al., 2021; Zeng et al., 2021), of which Q203 (Telacebec) is the most advanced through clinical trials (Pethe et al., 2013). Structural studies have demonstrated that Q203 binds to the menaquinone binding site, effectively blocking menaquinone oxidation (Zhou S. et al., 2021). This mechanism of action is supported by resistance against Q203 and the majority of other Cyt-*bc*_1_ *aa*_3_ inhibitors being at residues near the menaquinone-binding site (Liu et al., 2019; Lu et al., 2019; Yanofsky et al., 2021; Zhou S. et al., 2021).

Cytochrome bd-type menaquinol oxidase (Cyt-*bd*) is an alternative non-proton pumping high-affinity terminal oxidase that is only found in Prokaryotes (Borisov et al., 2021). From an energetic point of view, Cyt-*bd* is considered less efficient as it does not translocate protons. Despite this, Cyt-*bd* is able to generate a PMF by transmembrane charge separation with an H+/e ratio of 0.94 ± 0.18 (Borisov et al., 2011). Cyt-*bd* is encoded by *cydAB* as part of the *cydABDC* operon, with both genes being non-essential for mycobacterial growth (McNeil et al., 2021). Because of the reduced efficiency but higher affinity, Cyt-*bd* functions predominantly under low oxygen conditions *in vitro* (Kana et al., 2001; Berney and Cook, 2010; Aung et al., 2014) and *in vivo* (Shi et al., 2005; Cai et al., 2021). Cyt-*bd* becomes essential for mycobacterial growth when the Cyt-*bc*_1_ *aa*_3_ supercomplex is deleted or expression inhibited (Matsoso et al., 2005; Arora et al., 2014; Lu et al., 2015; Kalia et al., 2017; Lu et al., 2018; Beites et al., 2019; Lee et al., 2019; Bosch et al., 2021; Hards et al., 2022).

## Mycobacterial F_1_F_o_ ATP synthase

The PMF generated by the mycobacterial ETC is used by the F_1_F_o_-ATP synthase to generate ATP from ADP and inorganic phosphate. The mycobacterial F_1_F_o_-ATP synthase is encoded by the of *atpBEFHAGDC* operon, with all genes being essential for mycobacterial growth (Griffin et al., 2011; Zhang et al., 2012; Bosch et al., 2021; McNeil et al., 2021) even on fermentable carbon sources like glucose (Tran and Cook, 2005). Furthermore, the transcriptional repression of *atpE* and *atpB* not only inhibited mycobacterial growth, but resulted in rapid bacterial killing (McNeil et al., 2020). BDQ (Sirturo^®^) was approved by the US FDA in 2012 for the treatment of adults with pulmonary MDR-TB (Diacon et al., 2012; Cohen, 2013; Diacon et al., 2014). BDQ is generally bactericidal and can kill drug-resistant mycobacterial species and dormant bacilli (Diacon et al., 2012; Diacon et al., 2014). It acts quickly compared to most TB drugs, but still requires many weeks of therapy and BDQ-resistance has been reported, including in treatment-naïve populations (Andries et al., 2014; Villellas et al., 2017). BDQ targets the F_1_F_o_-ATP synthase of *M. tuberculosis* and binds to the *c*-subunit rotor in the membrane-embedded part of the F_1_F_o_-ATP synthase (Andries et al., 2005a; Preiss et al., 2015) decreasing intracellular ATP levels (Koul et al., 2007; Koul et al., 2008; Koul et al., 2014). Structural analysis demonstrated that BDQ binds to ATP synthase ‘leading’ and ‘lagging’ sites of subunit *c* and to a lesser degree subunit *a*, resulting in major conformational changes in mycobacterial ATP synthase (Guo et al., 2021; Montgomery et al., 2021). BDQ mediated inhibition of ATP synthesis also has downstream effects on a number of metabolic processes including the inhibition of glutamine synthetase (Wang et al., 2019) and the rerouting of mycobacterial metabolism that places an increased reliance on glycolysis and substrate level phosphorylation to generate cellular ATP (Mackenzie et al., 2020). BDQ also dissipates the ΔpH component of the PMF in mycobacteria (Hards et al., 2015; Hards et al., 2018). This depends on target-based accumulation of BDQ and leads to an uncoupled microenvironment around the F_1_F_o_-ATP synthase (Hards et al., 2018). Several new inhibitors of the mycobacterial F_1_F_o_-ATP synthase have been identified (Singh et al., 2015; Tantry et al., 2017; Narang et al., 2019; Kamariah et al., 2020; Hards et al., 2022). However, further studies are needed to determine their clinical safety and efficacy.

## Interactions with mycobacterial respiratory components

Mycobacterial respiratory components have emerged as a promising target space, not only for the development of novel therapeutics, but also as part of unique combination therapies. This is due to the observation that the inhibition of many mycobacterial respiratory components have positive interactions, not only with other respiratory components, but other cellular processes in general. For the remainder of this review, we describe each of these interactions and provide possible mechanistic explanations. We present interactions identified from either chemical, genetic and chemical-genetic interaction studies. We consider both (i) synergistic interactions (i.e. when the sum of the outcome is greater than the individual components) and (ii) synthetic lethal interactions, (i.e. when the loss/inhibition of neither component is lethal but combined results in cell death) as being “positive interactions”. We also discuss examples of antagonism, as identifying the basis for these unfavorable interactions will aid in the development of improved combination therapeutics. Whilst the following sections place an emphasis on interactions with clinically advanced inhibitors and their targets, i.e. BDQ, Q203 and pretomanid, we also discuss possible interactions between other respiratory components in the hope that this will highlight priority targets for future drug development.

## Interactions with the F_1_F_o_-ATP synthase and the inhibitor BDQ

Numerous inhibitors of the mycobacterial F_1_F_o_-ATP synthase have been identified (Singh et al., 2015; Tantry et al., 2017; Narang et al., 2019; Hotra et al., 2020; Kamariah et al., 2020; Denny, 2021; Choi et al., 2022; Hards et al., 2022), of which BDQ is the most clinically advanced and was FDA approved for the treatment of multi-drug resistant *M. tuberculosis* in 2012 (Diacon et al., 2012; Cohen, 2013; Diacon et al., 2014). The bioenergetic consequences of inhibiting the F_1_F_O_-ATP synthase have resulted in ATP synthase inhibitors, including BDQ, having favorable interactions with other respiratory inhibitors. For example, several studies have demonstrated interactions between simultaneous inhibition of menaquinone biosynthesis and ATP synthase function (Sukheja et al., 2017; Berube et al., 2019). Inhibitors of MenA, which catalyzes the penultimate step in menaquinone biosynthesis had a synergistic killing interaction with BDQ, resulting in complete sterilization of *M. tuberculosis* cultures within 14 – 21 days of treatment (Berube et al., 2019). The synergy between inhibition of menaquinone biosynthesis and the ATP synthase was not observed in genetic studies, although this may be attributable to variations in model organism (i.e. *M. smegmatis* vs *M. tuberculosis*) or experimental approach (i.e. genetic-genetic interactions vs chemical-chemical interactions) (Sukheja et al., 2017; Berube et al., 2019; McNeil et al., 2022).

The non-proton pumping type II NADH dehydrogenase (NDH-2) is essential for mycobacterial growth. Phenothiazine-like compounds including CFZ have long been thought to kill through a redox cycle involving reduction by NDH-2 and spontaneous re-oxidation by O_2_, producing toxic levels of ROS (Yano et al., 2006; Yano et al., 2011; Heikal et al., 2016). As BDQ stimulates the respiration rate of *M. tuberculosis* (Hards et al., 2015; Lamprecht et al., 2016; Hards and Cook, 2018; Hards et al., 2018), this results in an accumulation of reducing equivalents that potentiates CFZ reduction, increasing ROS production. This drives the rapid sterilization of *M. tuberculosis in vitro* and in macrophages when BDQ and CFZ are used in combination (Lamprecht et al., 2016; Berube and Parish, 2018). Genetic studies have also reported synergistic interactions between the mycobacterial NDH-2 and the ATP synthase (McNeil et al., 2022). CRISPRi knockdown of *ndh* and *atpE* in *M. smegmatis* is bactericidal by itself and thus prevents the identification of synergistic interactions (McNeil et al., 2022). McNeil et al. (2022) overcame this by engineering sub-optimal sgRNAs that produced a bacteriostatic phenotype. This allowed for the identification of interactions between genes that when knocked down in combination, resulted in cell death (McNeil et al., 2022). This multiplexed approach identified a synthetic lethal interaction between NDH-2 and the ATP synthase that killed *M. smegmatis* (McNeil et al., 2022). Further to this, an NDH-2 conditional mutant in *M tuberculosis* was also more sensitive to growth inhibition by BDQ (Beites et al., 2019).

Chemical and genetic interaction studies have failed to show an interaction between inhibition of the ATP synthase and primary terminal oxidase Cyt-*bcc-aa*_3_ (Lamprecht et al., 2016; Beites et al., 2019; McNeil et al., 2022). Despite this, deletion of the alternative terminal oxidase Cyt-*bd* has been shown to increase sensitivity of *M. tuberculosis* to killing by BDQ (Berney et al., 2014; Kalia et al., 2017). This interaction wasn’t reproduced in genetic studies, again possibly due to differences in experimental approach or model system (McNeil et al., 2022).

Given the cellular importance of ATP, it is surprising that following BDQ exposure metabolic remodeling allows mycobacterial to initially tolerate BDQ, with minimal killing observed in the first five days of exposure (Koul et al., 2014; Hards et al., 2015). However, disrupting the metabolic response to BDQ can remove this initial tolerance and sensitize *M. tuberculosis* to rapid killing. For example, substrate level phosphorylation is prioritized for the generation of ATP production following BDQ exposure (Mackenzie et al., 2020). As such genetic inhibition of glycolysis by deleting pyruvate kinase *pykA* increased BDQ lethality, sterilizing cultures within 5-6 days. Interestingly, this synergy between glycolysis and ATP synthase inhibition only occurred at specific glycolytic nodes, with deletion of the first rate-limiting site in glycolysis, i.e. phosphofructokinase not increasing BDQ lethality. BDQ exposure also has downstream effects on cellular glutamine levels, with the depletion of ATP limiting the ability of glutamine synthetase to convert glutamate into glutamine (Wang et al., 2019). Reduced glutamine levels suggested that BDQ treated cells would have increased susceptibility to the targeted inhibition of glutamine synthetase. Consequently, BDQ synergized with methionine sulfoximine, an inhibitor of glutamine synthetase resulting in increased BDQ lethality (Wang et al., 2019). BDQ has also been shown to synergize with inhibitors of mycobacterial stress response and cell wall synthesis (Lechartier et al., 2012; Wang et al., 2019; Yang et al., 2019).

The most promising combination therapy involving BDQ, is the BPaL regime, consisting of BDQ, pretomanid and linezolid. BPaL is an all oral regime, that when utilized over a 6 month period achieve 90% favorable outcomes in patients with extensively drug-resistant or non-responsive multi-drug resistant *M. tuberculosis* (Conradie et al., 2020). Whilst the BPaL regimen has since been FDA approved, BDQ has significant toxicity issues including hepatotoxicity, irregular cardiac rhythm, and increases in blood bilirubin (Moodliar et al., 2021; Yao et al., 2022). There are also concerns associated with BDQ resistance, which is the result of (i) mutations in the transcriptional regulator *Rv0678* that up-regulate the expression of the efflux pump MmpL5 or (ii) in residues of ATP synthase that prevent BDQ binding. Clinical resistance to BDQ *via* mutations in the transcriptional regulator *Rv0678* have already been reported, even in treatment naïve populations prior to the inclusion of BDQ (Huitric et al., 2010; Andries et al., 2014; Hartkoorn et al., 2014; Villellas et al., 2017).

Inhibitors of the mycobacterial F_1_F_o_-ATP synthase are promising anti-tubercular agents, particularly given their ability to synergize with a range of other inhibitors suggesting that they could form the backbone of novel therapeutic regimens. Whilst BDQ highlights the potential for this class of compound, issues with toxicity and resistance highlight the need for improved derivatives or completely new chemical classes of ATP synthase inhibitors.

## Interactions with cytochrome oxidase bcc-aa_3_ oxidase and the inhibitor Q203

*M. tuberculosis* respires oxygen *via* two terminal respiratory oxidases, Cyt-*bcc_-_aa*_3_ and Cyt-*bd* (Cook et al., 2017). Numerous small molecule inhibitors of the cytochrome *bc*_1_ complex have been identified (Abrahams et al., 2012; Pethe et al., 2013; Kang et al., 2014; Lu et al., 2019; Yanofsky et al., 2021; Zhou S. et al., 2021), of which Q203 (aka Telacebec) is the most advanced through clinical trials whilst TB47 is in preclinical development (Pethe et al., 2013; Kang et al., 2014; Lu et al., 2019). *M. tuberculosis* is able to control the flow of electrons to either oxidase providing metabolic flexibility when either oxidase is deleted or inhibited (Lamprecht et al., 2016). This has been demonstrated by the ability to obtain gene deletions of Cyt-*bcc_-_aa*_3_ or Cyt-*bd* independently, whilst the chemical or genetic inhibition of the Cyt-*bcc_-_aa*_3_ stops growth but the cells remain viable (bacteriostatic) (Beites et al., 2019; Bosch et al., 2021; McNeil et al., 2021; McNeil et al., 2022). Despite this, various studies have demonstrated that blocking this functional redundancy results in improved inhibitory outcomes. For example, chemical-genetic studies demonstrated that the deletion of one terminal oxidase, increases susceptibility to inhibition of the alternative, with mutants lacking Cyt-*bcc_-_aa*_3_ having increased susceptibility to inhibitors of Cyt-*bd* (Lu et al., 2018; Lee et al., 2021), and mutants lacking Cyt-*bd* having increased susceptibility to inhibitors of Cyt-*bcc_-_aa*_3_ (Arora et al., 2014; Lu et al., 2015; Kalia et al., 2017; Moosa et al., 2017; Liu et al., 2019; Lu et al., 2019; Chong et al., 2020). These positive interactions are supported by the observation that Q203 completely inhibits respiration in the Cyt-*bd* mutant (Lamprecht et al., 2016). Further to this, the simultaneous inhibition of both terminal oxidases has been shown to have synthetic lethal phenotypes, resulting in cell death (Kalia et al., 2017; Lu et al., 2018; Liu et al., 2019; Lee et al., 2021). Importantly, this bactericidal outcome of dual terminal oxidase inhibition is conserved when using diverse carbon sources under replicating conditions, under non-replicating conditions and in murine models of infection (Kalia et al., 2017; Lee et al., 2021; McNeil et al., 2022). Subsequently, this has spurred interest in identifying and developing improved inhibitors of Cyt-*bd*. Recent examples include ND-011992 (Lee et al., 2021), MQL-H2 (Harikishore et al., 2020) and HM2-16F (Hards et al., 2022), all of which have synthetic lethal interactions with Q203. The less-than-optimal pharmacokinetic properties of ND-011992 make it unsuitable for development and alternative chemical scaffolds targeting cytochrome *bd* are required (Lee et al., 2019). The recent high-resolution structures of cytochrome bd from *M. tuberculosis* and *M. smegmatis* now provide a detailed molecular framework for the discovery of new bd inhibitors (Safarian et al., 2021; Wang et al., 2021). Interestingly, in *Mycobacterium ulcerans*, which has a non-functional Cyt-bd and relies exclusively on Cyt-*bcc_-_aa*_3_ as a terminal oxidase, inhibitors of Cyt-*bcc_-_aa*_3_ are bactericidal (Scherr et al., 2018; Liu et al., 2019). Alternatively, naturally occurring polymorphisms in the Cyt-*bcc_-_aa*_3_ of *Mycobacterium abscessus* provide a high level of resistance to Q203 (Sorayah et al., 2019). Combined, this data demonstrates that starving *M. tuberculosis*, and potentially other mycobacterial species, of their ability to use oxygen as a terminal electron acceptor can result in bactericidal outcomes and is a vulnerable target for drug discovery (Bajeli et al., 2020; Lee B. S. et al., 2020; Sviriaeva et al., 2020).

Menaquinone is the primary electron carrier in *M. tuberculosis* (Dhiman et al., 2009; Debnath et al., 2012; Cook et al., 2017). Interestingly, the majority of Cyt-bc1aa3 inhibitors bind to the menaquinone binding site to block menaquinone oxidation (Lu et al., 2019; Yanofsky et al., 2021). Studies have demonstrated interactions between simultaneous inhibition of menaquinone biosynthesis and inhibition of Cyt-*bcc_-_aa*_3_ (Berube et al., 2019). Inhibitors of MenA, which catalyzes the penultimate step in menaquinone biosynthesis, synergized with a cytochrome *bc*_1_-*aa*_3_ inhibitor (Berube et al., 2019). The synergy between inhibition of menaquinone biosynthesis and the cytochrome *bc*_1_-*aa*_3_ terminal oxidase was further demonstrated in genetic studies, where the simultaneous transcriptional repression of *menD* and *qcrB* resulted in stronger growth inhibition than the single knockdown of either gene alone (McNeil et al., 2022). The inhibition of menaquinone biosynthesis not only reduces available menaquinone, but it is also likely to reduce the levels of intracellular ATP needed to sustain growth and survival (Dhiman et al., 2009; Sukheja et al., 2017). This coupled with the bioenergetic effects of Cyt-*bcc_-_aa*_3_ inhibitors that ultimately lead to a reduction in the intracellular levels of ATP would result in target synergy and bacterial cell death.

Several studies have also reported synergistic interactions between the mycobacterial NADH dehydrogenase type II enzymes (NDH-2) and the Cyt-*bcc_-_aa*_3_ terminal oxidase in mycobacteria (Beites et al., 2019; McNeil et al., 2022). CRISPRi knockdowns have uncovered a synthetic lethal interaction between NDH-2 and the Cyt-*bcc_-_aa*_3_ that killed *M. smegmatis* faster than the dual knockdown of *qcrB* and *cydB* on both fermentable and non-fermentable carbon sources (McNeil et al., 2022). Studies have also reported synergistic killing between CFZ, a proposed inhibitor of NDH-2 and inhibitors of Cyt-*bcc_-_aa*_3_ including Q203 and phenoxy alkyl benzimidazoles (PABs) under both replicating and non-replicating conditions (Lamprecht et al., 2016) (Berube and Parish, 2018). However, there are conflicting reports regarding this interaction as the deletion of *ctaE*-*qcrCAB* did not alter sensitivity to growth inhibition by NDH-2 inhibitors, including CFZ (Beites et al., 2019). It should be noted, that the same authors conclude that CFZ does not require NDH-2 to inhibit the growth of *M. tuberculosis* as conditional inactivation of NDH-2 (i.e. *ndhA* + *ndh* double mutant) in *M. tuberculosis* had no effect on the growth inhibitory properties of CFZ, (Beites et al., 2019). Between these various studies, differences in (i) biological readouts, i.e. bacterial viability vs growth inhibition, (ii) experimental approaches of gene deletion compared to gene knockdown and (iii) experimental model i.e. *M. tuberculosis* or *M. smegmatis* may go some way to explaining these reported differences. Despite this, we conclude that there is sufficient evidence to suggest that whilst targeting NDH-2 may not synergize with the growth inhibitory properties of Cyt-*bcc_-_aa*_3_ inhibition, they may offer another potential avenue to enhance the bactericidal activity of the normally bacteriostatic Cyt-*bcc_-_aa*_3_ inhibitors.

Cyt-*bcc_-_aa*_3_ inhibition also synergizes with the first line *M. tuberculosis* drugs PZA (PZA) and rifampicin (RIF) in a mouse model (Lu et al., 2019). Specifically, the Cyt-*bcc_-_aa*_3_ TB47 inhibitor exhibited a potent synergy with subtherapeutic doses of PZA and RIF, causing a 4- and 5-fold reduction in lung CFU compared to the respective monotherapy (Lu et al., 2019). Here, it was proposed that a disruption to the NADH/NAD+ ratio by TB47 may explain the synergy of TB47 with RIF and PZA. TB47 also exhibited a highly unique synergistic activity with clarithromycin *in vitro* and in mouse models against different mycobacterial species (Liu et al., 2020; Yu et al., 2020). TB47-containing 3-drug regimens cured Buruli ulcer *in vivo* in ≤ 2 weeks when dosed daily or in ≤ 3 weeks when dose twice per week (6 doses in total) compared to the current first-line treatment of RIF and streptomycin which required 8 weeks and had 26.67% mice relapsing post treatment (Gao et al., 2021).

The bioenergetic consequences of Cyt-*bcc_-_aa*_3_ inhibition, that lead to reduced ATP levels and severely impaired bacterial growth have also been shown to antagonise the bactericidal activities of the anti-tubercular drugs isoniazid (INH) and moxifloxacin (Lee et al., 2019). Specifically, the metabolic downregulation following Q203 exposure dissipated the transient increase in intracellular respiration that is associated with cell death and was observed following INH or moxifloxacin exposure alone (Lee et al., 2019). This is consistent with the antagonism frequently seen between bactericidal and bacteriostatic antibiotics (Ocampo et al., 2014). Interestingly, a known efflux pump inhibitor verapamil increased the potency of Q203 against *M. tuberculosis*, indicating that the upregulation of efflux pumps may be associated with low level resistance to Q203 (Jang et al., 2017). This further highlights how an improved understanding of the mechanisms of antibiotic mediated mycobacterial death are essential to the improved design of optimized combination therapies.

## Interactions between other mycobacterial respiratory components

There are many bioenergetic components in *M. tuberculosis* for which there are no bona-fide small molecule inhibitor with favorable pharmacological properties. Given the resource investment required to advance small molecule through the drug development pipeline, it is essential that high priority targets with the greatest chance of clinical success when used in combination therapies are identified and validated. Here we discuss interactions between other mycobacterial respiratory components, specifically NADH dehydrogenase, succinate dehydrogenase, malate dehydrogenase and menaquinone biosynthesis. We acknowledge that there may be is overlap with prior sections.

## NADH dehydrogenase inhibitor interactions

A synthetically lethal interaction exists between the two different NADH dehydrogenase enzymes (NDH-1 and NDH-2) in *M. tuberculosis*, with the deletion of NDH-2 (Δ*ndh*Δ*ndhA*) rendering *M. tuberculosis* susceptible to killing by NDH-1 inhibitors such as rotenone, which normally has no effect on the growth of *M. tuberculosis* (Beites et al., 2019). Of the two enzymes, NDH-2 is being actively pursued as a drug target as it is the primary mycobacterial NADH dehydrogenase (Weinstein et al., 2005; Shirude et al., 2012; Dunn et al., 2014; Harbut et al., 2018; Vilcheze et al., 2018; Beites et al., 2019) and is absent from mammalian mitochondria. To date, two main classes of compound interact with the mycobacterial NDH-2 enzymes: the phenothiazines [e.g., thioridazine (THZ), chlorpromazine (CPZ)] and CFZ, and both compounds have a range of positive interactions reported both *in vitro* and *in vivo*. For example, THZ and CPZ have been shown to enhance the activity of TB antibiotics including RIF and streptomycin (Crowle et al., 1992; Viveiros and Amaral, 2001).

While not technically an inhibitor of NDH-2, the mechanism of action of CFZ is linked to NDH-2 activity (Yano et al., 2011). CFZ is thought to kill through *via* a NDH-2 recycling mechanism that produces toxic levels of ROS (Yano et al., 2011). Accordingly, inhibitors that potentiate CFZ reduction and thus ROS production are synergistic with CFZ. Has highlighted from prior sections, this includes both BDQ and Q203 (Lamprecht et al., 2016). Inhibition of the Cyt*bc*_1_-*aa*_3_ oxidase with phenoxy alkyl benzimidazoles (PABs) synergized with CFZ under replicating and non-replicating conditions (Berube and Parish, 2018). These interactions are consistent with genetic studies, showing that the inactivation of *ndh* with either *atpE* or *qcrB* having a bactericidal phenotype (McNeil et al., 2022). Moreover, CFZ has been previously shown to directly compete with menaquinone for reduction by NDH-2 and, as such, menaquinone biosynthesis inhibitors synergize strongly with CFZ to kill *M. tuberculosis* (Berube et al., 2019). Interestingly, deletion of cytochrome *bd* synergized with NDH-2 inhibition in *M. tuberculosis* (Beites et al., 2019) and the deletion of cytochrome *bd* sensitized *M. smegmatis* to killing by CFZ at concentrations that were bacteriostatic against wild-type (Lu et al., 2015). This is despite no interaction being observed between the dual knockdown of *cydB* and *ndh* or *nuoD* in *M. smegmatis* (McNeil et al., 2022). These differences may be partly explained by choice of model system, experimental approach, or recent evidence suggesting that CFZ doesn’t require NDH-2 for inhibitory activity against *M. tuberculosis* (Beites et al., 2019). Further, studies in murine infection models have shown that CFZ synergizes with RIF, INH and PZA to halve the treatment period for drug-sensitive *M. tuberculosis* (Li et al., 2017) as well as potentiating the activity of second-line drug regimens against drug-resistant *M. tuberculosis* (Grosset et al., 2013). This suggests that targeting NDH-2 offers another potential avenue to enhance the activity of the normally bacteriostatic Cyt*bc*_1_-*aa*_3_ inhibitors although further work is required to reconcile some experimental differences.

While several promising synergies have been identified between inhibiting the mycobacterial NADH dehydrogenases and other respiratory complexes, it is also worth noting that there is also the potential for antagonistic interactions to occur, particularly between INH and CFZ. Mutations in *ndh* have previously been shown to confer INH resistance in *M. tuberculosis*, *M. smegmatis*, and *M. bovis* BCG (Miesel et al., 1998; Lee et al., 2001; Vilcheze et al., 2005), and both targeted protein depletion and chemical inhibition of NDH-2 attenuate INH activity in *M. tuberculosis* (Harbut et al., 2018; Koh et al., 2022). This antagonism is postulated to be due to alterations in the NADH/NAD^+^ ratio preventing the formation of the active INH-NAD adduct or competing with its binding to the NADH-dependent enoyl-ACP reductase, InhA (Miesel et al., 1998; Lee et al., 2001; Vilcheze et al., 2005; Koh et al., 2022). Despite this, genetic deletion of *ndh* and *ndhA* alone or in combination did not alter INH susceptibility in *M. tuberculosis*, which was attributed to insufficient NADH accumulation in these mutants to affect INH activity (Vilcheze et al., 2018; Beites et al., 2019). Nevertheless, future NDH-2 inhibitors should be investigated for their potential interactions with INH. Likewise, as CFZ is activated by NDH-2, interactions between NDH-2 inhibitors and CFZ should be evaluated. Importantly, two recent studies demonstrated that CFZ retains bactericidal activity against *M. tuberculosis* in the absence of NDH enzymes (Vilcheze et al., 2018; Beites et al., 2019), suggesting that antagonism with NDH-2 inhibitors is unlikely as other mechanisms of CFZ activation or activity exist independently of NDH-2.

## SDH and MQO/MDH inhibitor interactions

Whereas the NADH dehydrogenases inhibitor interactions have been well studied, the remaining electron-donating complexes are comparatively under studied and under explored as drug targets. A recent study showed that inhibition of SDH activity using CRISPRi sensitizes *M. tuberculosis* to growth inhibition and killing by BDQ and the cytochrome bcc-*aa*_3_ inhibitors, Q203 and TB47 (Adolph et al., 2022). Likewise, knockdown of *qcrB* or *ctaC* with *sdhA2* synergized in *M. smegmatis* (McNeil et al., 2022). Moreover, when gene knockdown was induced simultaneously with drug treatment, impaired succinate oxidation prevented the emergence of INH and PA-824(pretomanid) resistance, suggesting that SDH inhibitors may also be effective anti-resistance agents when used in combination with INH or pretomanid (Adolph et al., 2022). However, when SDH enzymes were pre-depleted prior to antibiotic challenge an antagonistic interaction was observed with both INH and pretomanid and the other cell wall inhibitors tested; ethionamide (ETH), EMB and SQ109 (Adolph et al., 2022). This is similar to the observations that BDQ and Q203 can attenuate the bactericidal activity of INH, EMB and ETH by preventing a drug-induced lethal ATP burst (Shetty and Dick, 2018; Lee et al., 2019; Zeng et al., 2021). Given the absence of specific SDH inhibitors, it remains to be determined which of these interactions are observed at the chemical level.

Inhibition of the two malate oxidizing enzymes, MDH and MQO, using CRISPRi synergizes to significantly impair the growth of *M. tuberculosis* compared to the knockdown of either gene alone (Harold et al., 2022). Interactions beyond this have yet to be investigated. However, it is possible that MDH or MQO inhibitors would synergize with NDH inhibitors as the net reaction of MQO and MDH acting in concert is the same as the NADH dehydrogenase reaction (Blaza et al., 2017), suggesting these three enzymes may be functionally redundant (Molenaar et al., 2000). Consistent with this, MDH was shown to be able to compensate for *ndh* mutants in *M. smegmatis* (Miesel et al., 1998; Vilcheze et al., 2005) and CRISPRi studies showed a weakly additive interaction between the knockdown of *ndh* and *mqo* in *M. smegmatis* (McNeil et al., 2022). However, these interactions require further investigation in *M. tuberculosis.*

## Menaquinone biosynthesis inhibitor interactions

Several studies have demonstrated that chemical or genetic inhibition of menaquinone biosynthesis sensitizes mycobacteria to further inhibition of various other respiratory complexes (Sukheja et al., 2017; Berube et al., 2019; McNeil et al., 2022). Specifically, inhibition of MenA, which catalyzes the penultimate step in menaquinone biosynthesis, synergized with sub-bactericidal concentrations of BDQ, CFZ and the cytochrome *bcc*-*aa*_3_ inhibitor, ND-10885, resulting in complete sterilization of *M. tuberculosis* cultures within 14 - 21 days of treatment (Berube et al., 2019). Likewise, inhibition of the terminal biosynthetic enzyme, MenG, with the biphenyl amide DG70 also synergized with BDQ, again sterilizing cultures within 21 days (Sukheja et al., 2017). DG70 displayed further synergy with pretomanid, sterilizing cultures of *M. bovis* BCG within 10 days, as well as having an additive interaction with the NDH-2 inhibitor, THZ (Sukheja et al., 2017).

As previously highlighted, the synergy between inhibition of menaquinone biosynthesis and the cytochrome *bc*_1_-*aa*_3_ terminal oxidase was genetically validated (McNeil et al., 2022). Interestingly, CRISPRi did not recreate the synergy between BDQ (*atpE* knockdown) and menaquinone biosynthesis inhibition (*menD* knockdown), and the synthetic lethality between *ndh* knockdown (representing CFZ and THZ interactions) and *menD* was only observed when glycerol was used as the primary carbon source (McNeil et al., 2022). This is likely due to insufficient gene knockdown using CRISPRi or metabolic buffering of residual menaquinone preventing an observable interaction (Donati et al., 2021; McNeil et al., 2022). Nevertheless, disruption of menaquinone biosynthesis appears to be broadly synergistic with other bioenergetic inhibitors in *M. tuberculosis*. Mechanistically, this may be explained by the fact that MenA and MenG inhibitors completely block mycobacterial oxygen consumption (Dhiman et al., 2009; Sukheja et al., 2017) and reduce intracellular ATP levels by > 50%, which may sensitize *M. tuberculosis* to further perturbations of ETC activity, resulting in lethal disruptions to ATP synthesis and PMF generation.

Beyond the respiratory chain, inhibition of MenG with DG70 has also been shown to synergize with RIF, as well as significantly enhancing the activity INH (Sukheja et al., 2017). The dual treatment of log phase cultures of *M. bovis* BCG with INH and DG70 rapidly increased the rate of killing compared to either drug alone and sterilized cultures within 10 days (Sukheja et al., 2017). As previously highlighted, inhibiting respiration by targeting the ATP synthase or cytochrome *bcc*-*aa*_3_ terminal oxidase prevents INH killing of *M. tuberculosis* and *M. bovis* BCG (Shetty and Dick, 2018; Lee et al., 2019; Zeng et al., 2021), raising concerns that bioenergetic inhibitors may have unintended antagonistic interactions with conventional TB drugs. However, the potent synergy between DG70 and INH demonstrates that this is not always the case and provides an alternative approach to retaining the benefits of bioenergetic inhibitors (i.e., bactericidal activity under replicating and non-replicating conditions) while avoiding antagonistic interactions with current TB drugs. Defining the mechanisms for these different interactions will allow for further improvements in the design of combination therapies involving bioenergetic inhibitors.

In addition to specific inhibitors of the menaquinone biosynthesis pathway, SQ109, which is undergoing clinical development for the treatment of drug-resistant *M. tuberculosis*, has also reported to target both MenA and MenG (Li et al., 2014), in addition to functioning as an uncoupler and MmpL3 inhibitor (Feng et al., 2015). SQ109 synergizes with INH and RIF *in vitro* and in chronic mice models of infection (Chen et al., 2006; Nikonenko et al., 2007) providing another example of potential synergies that can be achieved by targeting menaquinone biosynthesis. However, given the multiple mechanisms of action of SQ109, it is unclear to what degree inhibition of menaquinone biosynthesis contributes to this synergy.

Overall, menaquinone biosynthesis inhibitors offer a broad range of synergies and avoids antagonistic interactions seen by some bioenergetic inhibitors suggesting they could be efficacious in a broad range of regimens. Particularly promising is the ability to enhance the activity of currently available bioenergetic inhibitors, namely BDQ, pretomanid and terminal oxidase inhibitors Q203.

## Interactions with the F_420-_dependent respiratory inhibitor pretomanid

Pretomanid is a bicyclic nitroimidazole derivative that was discovered when testing 3-substituted bicyclic nitroimidazole-containing compounds for antitubercular activity (Stover et al., 2000; Manjunatha et al., 2006a; Manjunatha et al., 2009). Treatment with pretomanid *in vitro* is bactericidal at sub-micromolar concentrations in *M. tuberculosis* and it shows a narrow spectrum of activity that ranges from low to no activity in non-tubercular *Mycobacteria* (Stover et al., 2000; Manjunatha et al., 2006a; Manjunatha et al., 2006b; Manjunatha et al., 2009; Zheng et al., 2022). Whilst not a strict bioenergetic inhibitor, pretomanid kills both replicating as well as hypoxic non-replicating bacilli by targeting both energy production and cell wall synthesis of *M*. *tuberculosis* (Singh et al., 2008; Manjunatha et al., 2009). Catalysis of pretomanid from a prodrug to an active form is carried out by the F_420_-dependent nitroreductase Ddn (Manjunatha et al., 2006a; Manjunatha et al., 2009; Lee B. M. et al., 2020). Ddn is suggested to protect against oxidative stress by reduction of quinones to dihydroquinones, which avoids the cytotoxic effects of semiquinones (Gurumurthy et al., 2012; Gurumurthy et al., 2013). Resistance to pretomanid is through mutations in genes essential for F_420_ synthesis or recycling pathways including *ddn*, *fgd1*, or the *fbi* gene products (Lee B. M. et al., 2020; Gomez-Gonzalez et al., 2021), all of which are non-essential under replicating conditions (Griffin et al., 2011). No cross-resistance to bicyclic nitroimidazole has been observed with any other class of anti-tubercular drug (Stover et al., 2000; Matsumoto et al., 2006).

The bactericidal activity of pretomanid is driven by the respiratory poisoning and inhibition of mycolic acid synthesis (Stover et al., 2000; Singh et al., 2008). Transcriptional profiling of pretomanid-treated *M. tuberculosis* under aerobic conditions showed dysregulation of genes controlling cell wall synthesis such as the *iniBAC* operon, as well as genes that respond to respiratory poisoning such as the *cydABDC* operon (Boshoff et al., 2004). This respiratory poisoning has differing effects on replicating and non-replicating cells: non-replicating cells under hypoxic conditions are unable to exit dormancy and replicating cells are unable to synthesize the cell wall. Similar mechanisms of killing of pretomanid have been observed in studies of the FDA approved drug delamanid that is also a bicyclic nitroimidazole (Matsumoto et al., 2006; Xavier and Lakshmanan, 2014). Metabolomics of pretomanid-treated *M. smegmatis* showed the additional accumulation of phosphate sugars, consistent with reduced FGD1 activity in the pentose-phosphate pathway, leading to cell arrest through accumulation of the toxic metabolite methylglyoxal that modifies peptides and DNA (Murata-Kamiya and Kamiya, 2001; Thornalley et al., 2010; Baptista et al., 2018). This multi-target phenotype of pretomanid suggests that it has the potential to synergize with bioenergetic inhibitors in addition to inhibitors that target cell wall synthesis.

Pretomanid was FDA approved in August 2019 as part of the BPaL regime, an all oral treatment for extensively drug-resistant or treatment non-responsive multi-drug resistant tuberculosis (Keam, 2019). BPaL consists of BDQ, PMD, and linezolid, and in clinical trials showed a favorable results, with 90% of patients achieving a culture negative status (Conradie et al., 2020). The positive results of this combination suggests that pretomanid has positive interactions with either BDQ or linezolid. Potential synergy between BDQ and pretomanid has been identified in part through pretomanid-led repression of the transcription factor Rv0880 that activates a tolerance response and leads to survival against BDQ (Peterson et al., 2016). Multiple links between pretomanid-induced disruption of the stress response appear to underpin further synergistic interactions. This includes pretomanid-mediated reduction in the expression of dormancy genes, contributing to a reduced oxygen consumption and ATP levels of the bacilli leading to a synergistic interaction between the Cyt-bc1aa3 inhibitor TB47 (Zeng et al., 2021). The biphenyl amide DG70 that targets MenG required for menaquinone biosynthesis also showed enhanced bactericidal activity with pretomanid against *in vitro* cultures of *M. tuberculosis* (Sukheja et al., 2017).

Synergistic drug interactions with pretomanid have also been observed in mice with addition of PZA or moxifloxacin to combination of BDQ plus pretomanid showing reduced time to relapse compared to BDQ + pretomanid alone, and the combination of all four drugs showed a further decrease in relapse (Tasneen et al., 2011; Li et al., 2017). The addition of linezolid to BDQ plus pretomanid treatment of *M. tuberculosis* HN878 in mice showed lower efficacy compared to BDQ plus pretomanid alone but greater efficacy for the treatment of *M. tuberculosis* H37Rv. This highlights that differences between geographic lineages of *M. tuberculosis* may confound the efficacy of not only individual drugs but unique drug combinations (Bigelow et al., 2020).

## Review Conclusion

Inhibitors of mycobacterial bioenergetics have been shown to have significant clinical potential, in combating both the spread of drug resistance and in reducing treatment times for drug susceptible strains of *M. tuberculosis*. Much of this potential relies on the ability of metabolic inhibitors to disrupt mycobacterial metabolism and potentiate the activity of other anti-tuberculosis agents, in particular other inhibitors of mycobacterial bioenergetics or metabolism. Achieving the full clinical potential of bioenergetic combinations requires a thorough understanding of the consequences of inhibiting specific bioenergetic nodes, both in isolation and in combination with other metabolic dysregulation. Whilst significant insights have been gained from studying the effects of on-target chemical inhibitors, many potential drug targets lack bona-fide chemical inhibitors. Genetic tools to deplete essential genes at a transcriptional and/or a translation level have filled this gap and provided significant insights into the consequences of inhibiting mycobacterial bioenergetics as well as the potential for highly efficacious combination therapies. The continued advancement and application of the mycobacterial genetics should allow for the continued investigation and prioritization of drug targets and unique combination therapies centered around bioenergetic inhibitors. There is also a need for an improved understanding of the mechanisms that provide resistance to bioenergetic inhibitors, including those in pre-existing clinical isolates, as they will allow for the development of companion diagnostic tools to further the clinical efficacy and lifespan of these novel agents and combinations.

## Author contributions

All authors contributed to the writing and editing of this review.

## Funding

This work was supported by the Joint Research Health Research Council of New Zealand (HRC)-NSFC Collaboration grant numbers 20/1211 (to GC), 82061128001 (to TZ), and partially by the Chinese Academy of Sciences (154144KYSB20190005, YJKYYQ20210026). This work was also supported by Joint funding from the Maurice Wilkins Centre and the Chinese Academy of Sciences (awarded to MM, GC, and ZL). The funders had no role in study design, data collection, and analysis, decision to publish, or preparation of the manuscript.

## Conflict of interest

The authors declare that the research was conducted in the absence of any commercial or financial relationships that could be construed as a potential conflict of interest.

## Publisher’s note

All claims expressed in this article are solely those of the authors and do not necessarily represent those of their affiliated organizations, or those of the publisher, the editors and the reviewers. Any product that may be evaluated in this article, or claim that may be made by its manufacturer, is not guaranteed or endorsed by the publisher.
